# Isolation, abundance and phylogenetic affiliation of endophytic actinomycetes associated with medicinal plants and screening for their *in vitro* antimicrobial biosynthetic potential

**DOI:** 10.3389/fmicb.2015.00273

**Published:** 2015-04-07

**Authors:** Ajit K. Passari, Vineet K. Mishra, Ratul Saikia, Vijai K. Gupta, Bhim P. Singh

**Affiliations:** ^1^Molecular Microbiology and Systematics Laboratory, Department of Biotechnology, Mizoram UniversityAizawl, India; ^2^Biotechnology Division, CSIR-North East Institute of Science and TechnologyJorhat, Assam, India; ^3^Molecular Glyco-biotechnology Group, Department of Biochemistry, National University of Ireland GalwayGalway, Ireland

**Keywords:** endophytic actinomycetes, 16S rRNA gene, antibiotic sensitivity, polyketide synthase (PKS-I), non-ribosomal peptide synthetase (NRPS)

## Abstract

Microorganisms associated with medicinal plants are of interest as the producers of important bioactive compounds. To date, the diversity of culturable endophytic actinomycetes associated with medicinal plants is in its initial phase of exploration. In this study, 42 endophytic actinomycetes were isolated from different organs of seven selected medicinal plants. The highest number of isolates (*n* = 22, 52.3%) of actinomycetes was isolated from roots, followed by stems (*n* = 9, 21.4%), leaves (*n* = 6, 14.2%), flowers (*n* = 3, 7.1%), and petioles (*n* = 2, 4.7%). The genus *Streptomyces* was the most dominant among the isolates (66.6%) in both the locations (Dampa TRF and Phawngpuii NP, Mizoram, India). From a total of 42 isolates, 22 isolates were selected for further studies based on their ability to inhibit one of the tested human bacterial or fungal pathogen. Selected isolates were identified based on 16S rRNA gene analysis and subsequently the isolates were grouped to four different genera; *Streptomyce*s, *Brevibacterium*, *Microbacterium*, *and Leifsonia*. Antibiotic sensitivity assay was performed to understand the responsible antimicrobials present in the isolates showing the antimicrobial activities and revealed that the isolates were mostly resistant to penicillin G and ampicillin. Further, antimicrobial properties and antibiotic sensitivity assay in combination with the results of amplification of biosynthetic genes polyketide synthase (PKS-I) and non-ribosomal peptide synthetase (NRPS) showed that the endophytic actinomycetes associated with the selected medicinal plants have broad-spectrum antimicrobial activity. This is the first report of the isolation of *Brevibacterium* sp., *Microbacterium* sp., and *Leifsonia xyli* from endophytic environments of medicinal plants, *Mirabilis jalapa* and *Clerodendrum colebrookianum*. Our results emphasize that endophytic actinomycetes associated with medicinal plants are an unexplored resource for the discovery of biologically active compounds.

## Introduction

Endophytic microorganisms reside in the internal tissues, and subsist in symbiotic or mutualistic association with their host plant without causing apparent symptoms of infection. Endophytes are ubiquitous and present in almost all plant species on earth and moreover, it is commonly speculated that they contribute to the evolutionary fitness of their host by producing a range of secondary metabolites, which provide resistance against diseases and survival (Strobel et al., 2004). Actinomycetes are the most frequently isolated endophytes followed by gram-positive and gram-negative bacteria, explored as a novel source for the production of bioactive compounds. Actinomycetes are aerobic, gram positive bacteria comprising a group of branching unicellular microorganism, which play a significant role in the breakdown of organic matter into more easily obtainable nutrients. They are also known for the production of many secondary metabolites including various antibiotics, antitumor and plant growth hormones, which are important for pharmaceutical and agricultural industries (Fiedler et al., 2008).

Endophytic actinomycetes, recovered from healthy surface sterilized tissues in particular are considered as potential source for the production of secondary metabolites, various natural products with antimicrobial, antioxidants and plant growth promoting activities (Merckx et al., 1987; Lam, 2006; Nimnoi et al., 2010). There is increasing evidences for the existence of new endophytic actinomycetes within various tissues of medicinal plants, and some produce bioactive compounds with novel chemical structures (Qin et al., 2008; Nimnoi et al., 2010). However, information is scarce on the tissue distribution and biodiversity of endophytic actinomycetes associated with traditional medicinal plants from unique environments.

Screening of endophytic actinomycetes for their functional role is a promising approach to overcome the mounting threats of drug resistance against human and plant pathogens (Tan and Zou, 2001). It has been well documented that medicinal plants with an established ethnobotanical history are promising candidates for the isolation of potent endophytic microorganisms as these microorganism also plays a significant role in the development of medicinal property of the plants (Yu et al., 2010). For instance, Qin et al. (2009) isolated 46 antimicrobial endophytic actinomycetes from medicinal plants of tropical rain forest and they showed that medicinal plants are the reservoir of novel endophytic actinomycetes for the isolation of biologically active compounds. Recently, Zhao et al. (2011) also isolated 560 endophytic actinomycetes from medicinal plants of China and stated that isolates were displaying broad spectrum antimicrobial activity and proved that endophytic actinomycetes are an important pool for bioactive compounds.

Different ecological environments greatly influence the biological diversity and species distribution among the host plants (Sheil, 1999; Hou et al., 2009). Previous studies, have examined the diversity and antimicrobial potential from many medicinal plants from various parts of the world (Qin et al., 2009; Verma et al., 2009). In this study we isolated and characterize the diversity of endophytic actinomycetes and their biosynthetic potential associated with some ethnobotanical medicinal plants from Mizoram, Eastern Himalaya.

## Materials and methods

### Sampling of medicinal plants

**Figure 1:**
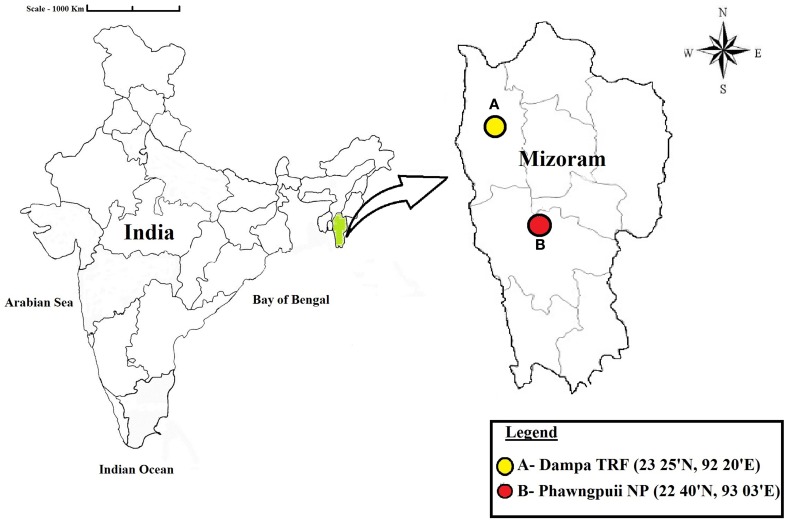
**Map showing the locations of the sampling sites**. Dampa Tiger Reserve forest and Phawngpuii National Park, Mizoram, India.

Healthy medicinal plants were collected from Phawngpuii National Park [Phawngpuii NP] (22°40′N; 93°03′E) and Dampa Tiger Reserve Forest [Dampa TRF] (23°25′N; 92°20′E) in Mizoram, India during November, 2012 (Figure 1). The distance between the selected sites is 242 miles. From seven selected plants, 560 tissues of leaf, stem, root, flower (if present) and petiole were taken. Roots were collected by digging the soil adjacent of the main stem and collecting samples about 0.6 cm diameter and 5–6 cm in length. The cut ends were sealed with wax, and then all samples were brought into the laboratory in an icebox and used for isolation within 48 h.

### Isolation of endophytic actinomycetes and relative abundance

Collected plant materials were separated into individual organs i.e., leaves, stems, roots, flowers, and petioles by cutting with a scalpel. All samples were washed carefully in tap water for 3–5 min to remove soil and organic debris, and successively cut into pieces of 1 cm^2^, rinsed in 0.1% Tween 20 for a few seconds, and transferred to clean conical flasks. Samples were surface sterilized by immersing them sequentially in 70% ethanol for 3 min, followed by 0.4% NaOCl for 1 min, 70% ethanol for 2 min, followed by three washes with sterile distilled water for 1 min each. Samples were dried in a laminar airflow chamber and by using sterile scalpel, outer tissues were removed; the inner tissue of 0.5 cm size was carefully dissected. It was placed on Petri dishes containing different isolation media supplemented with nystatin and cycloheximide (60 μg/ml) to suppress fungal growth. The media was amended with nalidixic acid and K_2_Cr_2_O_7_, both in a final concentration of 60 μg/ml to restrict the grown of non-actinomycetes. The plates were incubated at 26 ± 2°C in BOD incubator for 2–4 weeks. Bacterial colonies growing out of the plated tissue segments were transferred onto ISP2 media slants and repeatedly recultured until pure cultures were obtained, and maintained at 4°C.

### Isolation media and conditions

The following five isolation media were used, all supplemented with 2.0% agar, nystatin, and cycloheximide to suppress fungal growth, nalidixic acid and K_2_Cr_2_O_7_ to inhibit fast growing bacteria, all in final concentration of 60 μg/ml: 1. Starch Casein Nitrate Agar (SCNA); 2. Actinomycetes Isolation Agar (AIA); 3. Tap Water Yeast Extract Agar (TWYE); 4. Yeast Malt Extract Agar (ISP2); 5. Glycerol Asparagine Agar (ISP5) as described by Taechowisan and Lumyong (2003).

To validate the efficacy of surface sterilization and to prove that the isolates indeed emerged from internal tissues of the host plants fingerprints of the surface sterilized tissues were taken on ISP2 agar media plates and incubated at 26 ± 2°C, and in addition, surface sterilized tissues were washed in sterilized distilled water at least three times, soaked in water for 1 min with continuous stirring. A 0.1 ml aliquot of the last wash was inoculated again on ISP2 media. If no microbial growth was observed on the agar plates, the sterilization was considered as effective (Schulz et al., 1993).

### Preliminary identification of actinomycetes

Analysis of the morphological and cultural characteristics of endophytic actinomycetes was carried out according to the International *Streptomyces* Project (Shirling and Gottlieb, 1966). All the isolates were identified up to genus level; including color of aerial and substrate mycelium, color and characteristics of the colony on the Petri plate, spore mass color, production of diffusible pigment, utilization of carbon sources and spore chain morphology for identification up to genus level (Goodfellow and Haynes, 1984). The structure of mycelium was observed using an oil immersion microscope. The spore chain morphology and surface of spore were examined by Field Emission Gun—Scanning electron microscopy (FEG-SEM) of 10-day old cultures grown on ISP1 media. The organism was identified by following the keys of Bergey's Manual of Determinative Bacteriology (Bergey and Holt, 2000).

### Screening for antimicrobial activity

Antimicrobial screening was performed against *Staphylococcus aureus* (MTCC-96), *Pseudomonas aeruginosa* (MTCC-2453), *Escherichia coli* (MTCC-739), and *Candida albicans* (MTCC-3017). The cultures were obtained from Microbial Type Culture Collection (MTCC), Chandigarh, India. Endophytic actinomycetes were inoculated in Tryptone yeast extract broth medium (ISP medium 1) and incubated at 28°C, 250 rpm for 7–10 d. Cells were harvested by centrifugation at 8000 rpm and the supernatant was collected into a fresh tube and tested for antimicrobial activity by the agar well diffusion method (Saadoun and Muhana, 2008). The test pathogenic microbes were inoculated on nutrient agar plate and wells of 6 mm diameter were prepared by using sterile cork borer. In each of the plates, wells were filled with 50 μl of clear supernatant of endophytic actinomycetes and the plates were incubated at 28 ± 2°C for 24 h. All experiments were performed in triplicates.

### Antibiotic sensitivity test

Ten different standard antibiotic discs were used against endophytic actinomycetes isolates to check the antibiotic sensitivity pattern on Muller Hinton agar medium. The isolates were inoculated in ISP 2 broth and incubated at 28 ± 2°C, 250 rpm for 7–10 d. The grown cultures were distributed with a sterile spreader over the plates of Muller Hinton agar. The antibiotic discs were placed on the plate and incubated at 28 ± 2°C for 24 h. All experiments were performed in triplicate. Antibiotic sensitivity was observed by measuring inhibition zone diameters as described earlier (Williams et al., 1989). Actinomycetes isolates were either considered as sensitive (S), intermediate (I), or resistant (R) to an antibiotic.

### DNA isolation, 16S rRNA gene amplification and phylogenetic analysis

Genomic DNA was extracted using FastPrep kit (MP Biomedical, USA) according to the manufacturer's protocol. All the isolates were subjected to the amplification of 16S rRNA gene by using universal primers (forward 16S rRNA primer 5′-AGAGTTTGATCCTGGCTCA-3′ and reverse 16S rRNA primer 5′-ACGGCTACCTTGTTACGACT-3′) (Cui et al., 2001). Reactions were performed in a Veriti thermal cycler (Applied Biosystem, Singapore) in a total volume 25 μl consisting of 1.0 μl genomic DNA (50 ng), 0.5 μl of each primer (10 pmol), 2.0 μl of deoxynucleotide triphosphates (2.5 mM each), 2.5 μl of 1x PCR buffer, 1.0 μl of Taq DNA polymerase (1 U/μl) and 17 μl MilliQ grade water. PCR was performed under the following conditions: initial denaturation step at 95°C for 4 min, followed by 30 cycles of denaturation at 94°C for 1 min, annealing at 57°C for 1 min and extension at 72°C for 1.2 min with a final extension step at 72°C for 10 min. A negative control reaction mixture without DNA template of actinomycetes was also included with each set of PCR reactions. The amplified PCR products were analyzed by electrophoresis through 1.2% agarose gels made in TAE buffer. The PCR bands were analyzed under UV light and documented using a Bio-rad Gel Doc XR^+^ system (Hercules, CA, USA). The PCR products were purified using QIAquick gel extraction kit (Qiagen, Hilden, Germany), and sequencing was done commercially at SciGenome Pvt. Ltd. Kochin, India.

DNA Sequence data were compared with GenBank/EMBL/DDBJ database using BlastN search program and sequences were aligned using Clustal W (Thompson et al., 1997). The evolutionary models were selected according to the lowest BIC (“Bayesian Information Criterion”) and AIC (“Akaike Information Criterion”) values using MEGA 5.05. Analysis based on 16S rRNA gene sequences used the model T92+ G (*G* = 0.21 and *R* = 0.42) and model K2+I (*I* = 0.55, *R* = 1.93) for construction of neighbor-joining and maximum likelihood trees, respectively. Phylogenetic trees were constructed using Tamura 3-parameter and Kimura 2 parameter models respectively with MEGA 5.05 (Saitou and Nei, 1987; Tamura et al., 2011), taking *E. coli* as an out group. The robustness of the phylogenetic trees was evaluated by bootstrap analysis with 1000 resamplings using *p*-distance model (Felsenstein, 1985). Trees were viewed and edited by using program FigTree 1.3.1 (2012).

### PCR amplifications of biosynthetic genes (PKSI and NRPS)

Polyketide synthase (PKS) type I gene fragments were amplified by using degenerate primers: K1F 5′-TSAAGTCSAACATCCGBCA-3′ and M6R 5′-CGCAGGTTSCSGTACCAGTA-3′ and non-ribosomal peptide synthetase (NRPS) gene fragments were amplified by using degenerate primers: A3F 5′-GCSTACSYSATSTACACSTCSGG-3′ and A7R 5′-SASGTCVCCSGTSGCGTAS-3′ (Ayuso-Sacido and Genilloud, 2005). The reaction was carried out in the Veriti thermal cycler (Applied Biosystems, Singapore) in a final volume of 50 μl containing 50 ng of genomic DNA, 2.0 U of Taq DNA polymerase, 1 mM MgCl_2_, 0.5 mM of dNTPs, 2.0 μM of each primer and 10% DMSO. PCR conditions consisted of one denaturation step at 96°C for 5 min, followed by 35 cycles of denaturation at 96°C for 60 s, annealing at 59°C for 60 s, and extension at 72°C for 2 min. Final extension step was done at 72°C for 10 min. A negative control reaction mixture without DNA template of actinomycetes was also included with each set of PCR reactions. The PCR products were visualized as stated above.

### Statistical analysis

The data (expressed as the mean ± standard deviation of mean of three replicates) were calculated using Microsoft Excel XP 2007 and One-Way analysis of variance (ANOVA) was performed to analyzed significant differences (*P* = 0.05) between antimicrobial activities of different isolates by using SPSS software version 20.0. Relative abundance of actinomycetes isolates was compared between the selected two locations by using Sigma Plot 12.0.

## Results

### Isolation, distribution and relative abundance of endophytic actinomycetes

**Figure 2:**
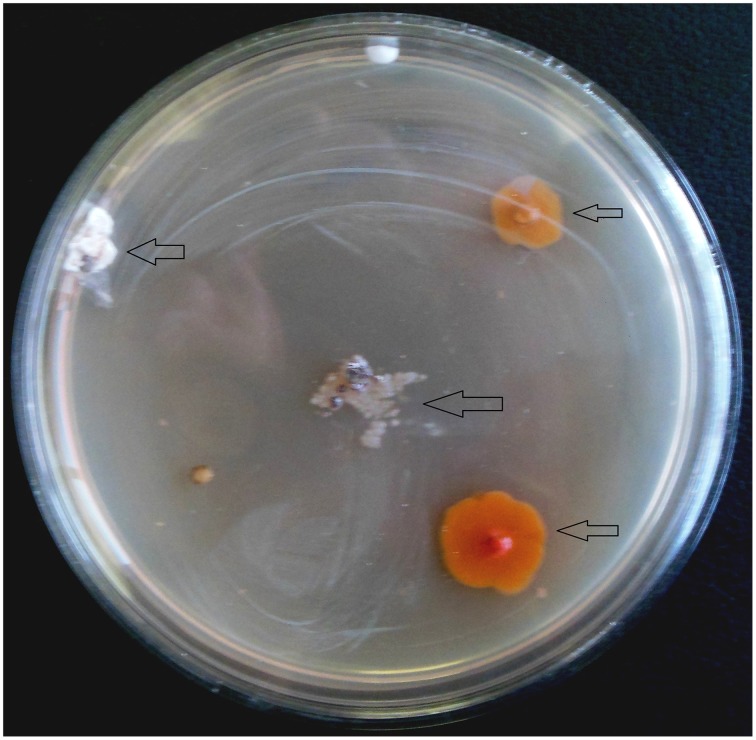
**Appearance of actinomycetes like colonies emerging from the organs of the plant after 3 weeks of incubation**. Arrows indicated the isolates.

**Figure 3:**
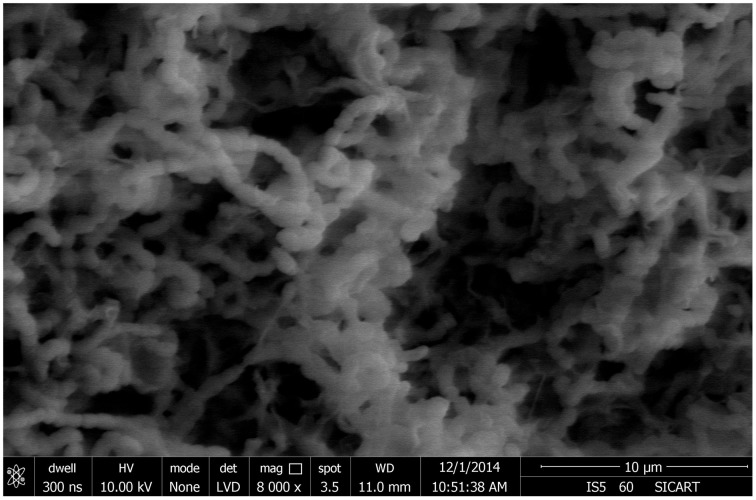
**Scanning electron microscope showing spore chain morphology of BPSAC38 isolate**.

From 560 tissues, 42 presumed endophytic actinomycetes were isolated and were further characterized based on colonial morphology, ability to form aerial hyphae and substrate mycelia. Out of 42 isolates, the majority (*n* = 22, 52.38%) were isolated from roots followed by stem (*n* = 9, 21.42%), leaf (*n* = 6, 14.28%), flower (*n* = 3, 7.14%), and petiole (*n* = 2, 4.76%). Thirteen isolates were from starch casein nitrate agar (SCNA) medium, 10 isolates from actinomycetes isolation agar (AIA) medium, 10 from tap water yeast extract agar (TWYE) medium, 6 from glycerol asparagines agar medium (ISP5) and 3 isolates were obtained from malt yeast extract agar medium (ISP2). Most of the isolates showed moderate to slow growth on the media. After 1 month incubation, the colonies of the actinomycetes were observed with white, yellow, orange, brownish white and pale yellow colors (Table 1 and Figure 2). The Field Emission Gun—Scanning electron microscopy (FEG-SEM) result showed that the aerial mycelia produce spiral spore chains (Figure 3). Most of the tissues except flower and petiole in some cases yielded at least one isolate which indicates that endophytic actinomycetes isolates can colonize different tissues throughout the plants.

**Table 1:** **Morphological and microscopic characteristics of endophytic actinomycetes isolates with their respective media and place of isolation**.

**Isolate No. and NCBI Genbank accession No.**	**Isolate identified as**	**Growth and colony nature**	**Aerial mycelia**	**Substrate mycelia**	**Pigmentation**	**Media name**	**Place**
BPSAC1	*Streptomyces* sp.	Slow and rough	White	White	No	SCNA	Phawngpuii NP
BPSAC2 (KF255557)	*Streptomyces* sp.	Slow and powdery	Brownish white	Light brown	No	SCNA	Dampa TRF
BPSAC3	*Microbacterium* sp.	Slow and smooth	Yellow	Light yellow	No	ISP5	Phawngpuii NP
BPSAC4	*Streptomyces* sp.	Slow and rough	Orange	Light orange	No	AIA	Dampa TRF
BPSAC5 (KF255560)	*Streptomyces* sp.	Slow and powdery	Brownish white	Brown	Yellowish brown	SCNA	Dampa TRF
BPSAC6	*Microbacterium* sp.	Slow and sticky	Orange	Light orange	No	SCNA	Phawngpuii NP
BPSAC7	*Streptomyces* sp.	Slow and rough	Brownish white	Brownish white	No	ISP5	Phawngpuii NP
BPSAC8	*Streptomyces* sp.	Slow and firm	Gray	Light brown	No	SCNA	Dampa TRF
BPSAC9	*Microbacterium* sp.	Slow and sticky	Orange	Orange	No	SCNA	Dampa TRF
BPSAC10	*Streptomyces* sp.	Slow and rough	Brownish white	Brownish white	No	ISP5	Dampa TRF
BPSAC11	*Actinomycete*	Slow and powdery	White	White	No	SCNA	Dampa TRF
BPSAC12	*Microbacterium* sp.	Slow and smooth	Yellow	Light yellow	No	SCNA	Dampa TRF
BPSAC13	*Streptomyces* sp.	Slow and rough	Brownish white	Brownish white	Yellowish brown	TWYE	Dampa TRF
BPSAC14	*Streptomyces* sp.	Slow and firm	Brownish white	Light brown	No	AIA	Phawngpuii NP
BPSAC15	*Streptomyces* sp.	Slow and powdery	Light brown	Brown	No	TWYE	Phawngpuii NP
BPSAC16	*Microbacterium* sp.	Slow and sticky	Yellow	Yellow	No	TWYE	Dampa TRF
BPSAC17	*Actinomycete*	Slow and rough	Cream white	Cream white	Light brown	ISP2	Dampa TRF
BPSAC18	*Streptomyces* sp.	Slow and rough	Brownish white	Brown	Light brown	AIA	Dampa TRF
BPSAC19	*Streptomyces* sp.	Slow and powdery	Gray	Light brown	Light brown	AIA	Dampa TRF
BPSAC20	*Streptomyces* sp.	Slow and firm	Light brown	Light brown	No	TWYE	Dampa TRF
BPSAC21 (KF255576)	*Microbacterium* sp.	Slow and sticky	Yellow	Yellow	No	SCNA	Dampa TRF
BPSAC22	*Streptomyces* sp.	Slow and rough	White	White	No	AIA	Phawngpuii NP
BPSAC23	*Streptomyces* sp.	Slow and powdery	Light brown	Light brown	No	TWYE	Phawngpuii NP
BPSAC24 (KJ584866)	*Leifsonia xyli*	Slow and firm	White	White	No	ISP5	Phawngpuii NP
BPSAC25 (KJ584867)	*Streptomyces* sp.	Slow and rough	Light brown	White	No	SCNA	Phawngpuii NP
BPSAC26 (KJ584868)	*Streptomyces* sp.	Slow and firm	Brownish white	Brown	Light brown	AIA	Phawngpuii NP
BPSAC27 (KJ584869)	*Microbacterium* sp.	Moderate and smooth	Orange	Light orange	No	TWYE	Phawngpuii NP
BPSAC28 (KJ584870)	*Microbacterium* sp.	Slow and smooth	Orange	Dark orange	No	TWYE	Phawngpuii NP
BPSAC29 (KJ584871)	*Microbacterium* sp.	Moderate and smooth	Orange	Dark orange	No	TWYE	Phawngpuii NP
BPSAC30 (KJ584872)	*Streptomyces olivaceus*	Slow and powdery	Brownish white	Light brown	No	SCNA	Phawngpuii NP
BPSAC31 (KJ584873)	*Streptomyces thermocarboxydus*	Slow and firm	Brownish white	Brown	No	AIA	Phawngpuii NP
BPSAC32 (KJ584874)	*Streptomyces* sp.	Slow and firm	White	White	No	TWYE	Phawngpuii NP
BPSAC33 (KJ584875)	*Streptomyces* sp.	Slow and firm	Gray	Blackish white	No	TWYE	Dampa TRF
BPSAC34 (KJ584876)	*Streptomyces* sp.	Moderate and firm	White	White	No	AIA	Dampa TRF
BPSAC35 (KJ584877)	*Brevibacterium* sp.	Moderate and smooth	Orange	Light orange	No	AIA	Dampa TRF
BPSAC36 (KJ584878)	*Streptomyces thermocarboxydus*	Slow and powdery	Brownish white	White	No	ISP5	Dampa TRF
BPSAC37 (KJ584879)	*Actinomycete*	Slow and smooth	Cream white	White	No	AIA	Dampa TRF
BPSAC38 (KJ584880)	*Streptomyces thermocarboxydus*	Moderate and firm	Brownish white	White	No	ISP5	Dampa TRF
BPSAC39 (KJ584881)	*Streptomyces* sp.	Slow and firm	Gray	Brown	No	SCNA	Dampa TRF
BPSAC40 (KJ584882)	*Streptomyces mutabilis*	Slow and firm	Brownish white	Brown	Dark brown	SCNA	Phawngpuii NP
BPSAC41 (KJ584883)	*Streptomyces* sp.	Slow and firm	Brownish gray	Brown	Dark brown	ISP2	Dampa TRF
BPSAC42 (KJ584884)	*Streptomyces mutabilis*	Slow and firm	Brownish gray	Brown	Dark brown	ISP2	Dampa TRF

SCNA, starch casein nitrate agar medium; AIA, Actinomycetes isolation agar medium; TWYE, tap water yeast extract agar medium; ISP5, glycerol asparagines agar medium; ISP2, malt yeast extract agar medium; Dampa TRF, Dampa Tiger Reserve Forest; Phawngpuii NP, Phawngpuii National Park.

**Figure 4:**
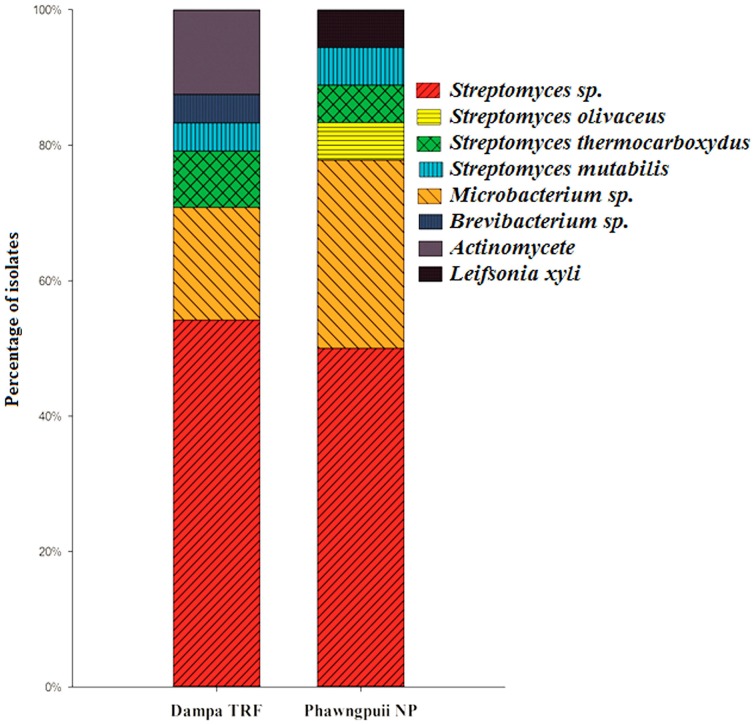
**The relative abundance of endophytic actinomycetes at the species level from Dampa TRF and Phawngpuii NP**.

Relative abundance of endophytic actinomycetes at the species level reveals that *Streptomyces* sp. was most abundant at Dampa TRF and Phawngpuii NP with 54.2 and 50.3%, respectively. *Microbacterium* sp. was dominant in Phawngpuii NP (27.7%) as compared to Dampa TRF (16.6%). However, some rare isolates like *Leifsonia xyli* recovered from Phawngpuii NP and *Brevibacterium* sp. along with *Actinomycete* were obtained from Dampa TRF (Figure 4). These results indicate that the population of endophytic actinomycetes varies between places likely influenced by different climatic conditions.

### Evaluation of antimicrobial activity

All 42 isolates were tested for antimicrobial activities against three bacterial pathogens *P. aeruginosa*, *S. aureus*, *E. coli*, and yeast *C. albicans*. Out of 42 isolates, 22 (52.3%) exhibited antagonistic activity against at least two of the four tested pathogens and all of them were positive against *S. aureus* and *E. coli* (Table 2). About two-third of isolates, inhibited the growth of all tested pathogens (Table 2). Among them, 13 isolates belongs to genus *Streptomyces*, two isolates belong to *Microbacterium* and one isolate each assigned under *Leifsonia* and *Brevibacterium* as designated by both morphological characteristics and 16S rRNA gene sequence analysis. Isolates BPSAC26 (*Streptomyces* sp.), BPSAC35 (*Brevibacterium* sp.), and BPSAC38 (*Streptomyces thermocarboxydus*) exhibited broad spectrum antimicrobial activities and are considered the most promising isolates for further attention. BPSAC38 showed activity against *S. aureus* (14.8 mm) and *E. coli* (9.8 mm), antimicrobial activity of BPSAC26 was also found to be high against *S. aureus* (12.8 mm) and *E. coli* (8.6 mm), BPSAC35 which is considered as a rare isolate showed the highest antimicrobial activity against *S. aureus* (13.7 mm) and *P. aeruginosa* (10.1 mm); whereas, different isolates of *Streptomyces* sp. showed an array of activity against bacterial and yeast pathogens, especially BPSAC39 (10.6 mm) and BPSAC37 (9.6 mm), had acute activities against *P. aeruginosa* and *C. albicans* respectively (Table 2).

**Table 2:** **Antibiotic sensitivity pattern, presence of biosynthetic genes (PKS1 and NRPS) and antimicrobial activity of the representative strains of endophytic actinomycetes**.

**Isolate No**	**Antimicrobial activity (zone of inhibition in mm)**				**Antibiotic sensitivity**										**Biosynthetic genes**	
	***P. aeruginosa***	***S. aureus***	***E. coli***	***C. albicans***	**G**	**P**	**V**	**N**	**T**	**Na**	**A**	**C**	**E**	**S**	**PKSI**	**NRPS**
BPSAC2	0.00^a^	6.6 ± 0.28^a^	4.0 ± 0.00^a^	0.00^a^	S	R	I	S	S	I	R	S	S	I	+	+
BPSAC5	0.00^a^	9.2 ± 0.57^bc^	6.3 ± 0.54^bc^	0.00^a^	S	R	I	S	S	R	R	S	S	I	−	−
BPSAC21	0.00^a^	7.0 ± 0.25^a^	4.0 ± 0.00^a^	0.00^a^	S	R	I	I	S	R	R	S	S	I	−	−
BPSAC24	6.9 ± 0.20bc	10.3 ± 0.57^bde^	4.3 ± 0.57^a^	7.1 ± 0.25^bc^	S	R	R	S	S	R	R	S	S	I	−	+
BPSAC25	5.3 ± 0.57^bde^	9.3 ± 0.57^bc^	4.3 ± 0.57^a^	6.3 ± 0.57^bde^	I	R	I	S	S	R	R	S	S	I	−	−
BPSAC26	5.1 ± 0.76^bde^	12.8 ± 0.76^bdfg^	7.6 ± 0.55^bde^	7.5 ± 0.10^bc^	I	R	R	I	S	R	R	S	S	R	+	+
BPSAC27	5.3 ± 0.57^bde^	9.3 ± 0.57^bc^	6.3 ± 0.57^bc^	5.1 ± 0.76^bdfg^	S	R	I	I	S	R	R	I	S	I	−	−
BPSAC28	0.00^a^	9.3 ± 0.57^bc^	9.6 ± 0.57^bdfg^	0.00^a^	S	R	I	S	S	I	R	I	S	R	−	−
BPSAC29	4.6 ± 0.57^bdfg^	7.1 ± 0.25^a^	9.6 ± 0.57^bdg^	5.1 ± 0.76^bdfg^	S	R	I	S	S	R	R	S	S	I	−	−
BPSAC30	6.3 ± 0.57^bc^	11.5 ± 0.50^bdfhi^	9.0 ± 1.0^bdfg^	5.9 ± 0.10^bdfhi^	S	R	I	S	S	R	R	S	S	R	−	+
BPSAC31	5.1 ± 0.76^bde^	11 ± 1.0^bdfhi^	4.0 ± 1.0^a^	4.0 ± 1.0^bdfhjk^	S	R	I	S	S	R	R	S	S	R	−	−
BPSAC32	0.00^a^	0.00^bdfhjk^	6.0 ± 0.64^bc^	5.5 ± 0.10^bdfg^	S	R	I	S	S	R	R	I	S	I	+	+
BPSAC33	6.2 ± 0.20^bdfhi^	12.3 ± 0.30^bdfg^	7.1 ± 0.25^bde^	4.0 ± 1.0^bdfhjk^	I	R	R	I	S	I	R	I	S	R	+	+
BPSAC34	8.0 ± 0.28^bdfhjk^	11.5 ± 0.50^bdfhi^	6.3 ± 0.57^bc^	5.1 ± 0.76^bdfg^	I	R	I	S	S	R	R	I	S	R	+	+
BPSAC35	10.1 ± 0.36^bdfhjlm^	13.7 ± 0.25^bdfhjlm^	6.3 ± 0.28^bc^	4.6 ± 0.57^bdfg^	S	R	R	I	S	R	R	I	S	R	+	+
BPSAC36	9.6 ± 0.57^bdfhjlm^	10.6 ± 0.57^bde^	4.5 ± 0.50^a^	5.1 ± 0.76^bdfg^	S	R	I	S	S	I	R	S	S	R	+	+
BPSAC37	4.6 ± 0.57^bdfg^	11.5 ± 0.50^bdfhi^	8.3 ± 0.26^bdfhi^	9.6 ± 0.57^bdfhjlm^	S	R	I	S	S	R	R	I	S	I	+	+
BPSAC38	6.2 ± 0.20^bdfhi^	14.8 ± 0.20^bdfhjln^	9.0 ± 1.0^bdfg^	8.0 ± 0.28^bdfhjln^	S	R	R	I	S	R	R	S	S	R	+	+
BPSAC39	9.6 ± 0.57^bdfhjlm^	13.4 ± 0.05^bdfhjlm^	7.1 ± 0.25^bde^	6.2 ± 0.20^bde^	S	R	R	S	S	I	R	I	S	R	+	+
BPSAC40	0.00^a^	11.2 ± 0.57^bdfhi^	6.3 ± 0.57^bc^	4.0 ± 1.0^bdfhjk^	I	R	I	S	S	R	R	I	S	R	−	−
BPSAC41	7.1 ± 0.25^bc^	7.2 ± 0.30^a^	4.6 ± 0.28^a^	6.2 ± 0.20^bde^	S	R	I	I	S	R	R	S	S	R	−	−
BPSAC42	0.00^a^	6.8 ± 0.05^a^	5.3 ± 0.26^bdfhj^	0.00^a^	S	R	R	S	S	R	R	S	S	R	+	+

Mean (±SD) followed by the same letter(s) in each column are not significantly different at P < 0.05 using Duncan's new multiple range test. Degree of susceptibility: >10 mm—Sensitive; 5.0–9.9 mm—intermediate); 0.0–4.9 mm—resistant. G, gentamicin (10 μg); P, Penicillin G (10 μg); V, vancomycin (30 ug); N, Norfloxcin (30 μg); T, tetracycline (30 μg); Na, Nalidixic acid (30 μg); A, ampicillin (10 μg); C, chloramphenicol (30 μg); E, erythromycin (15 μg); S, streptomycin (30 μg).

### Antibiotic sensitivity assay

To confirm the potency of the isolates as a source of new antibiotics, isolates were screened for their antibiotic sensitivity pattern against 10 standard antibiotics viz. gentamycin (G), penicillin G (P), vancomycin (V), norfloxicin (N), tetracycline (T), nalidixic acid (Na), ampicillin (A), chloramphenicol (C), erythromycin (E), and streptomycin (S). Most of the isolates showed high sensitivity against tetracycline and erythromycin (100% each) followed by gentamicin (77%), norfloxcin (68%) and chloramphenicol (59%). All isolates were resistance to penicillin G and ampicillin (100% each) whereas the degree of resistance to nalidixic acid, streptomycin and vancomycin was shown as 77, 59, and 31%, respectively (Table 2). The isolates BPSAC35, BPSAC38, and BPSAC26 showed resistance against 5 out of 10 antibiotics assessed; those might be a candidate for the discovery of antibiotics.

### Detection of PKS and NRPS genes in selected strains

The presence of genes encoding PKSI and NRPS were detected in 22 isolates of endophytic actinomycetes using two sets of degenerate primers. PCR amplification from 10 isolates out of 22 showed a band of the expected size for PKS type I (45%), whereas NRPS candidate amplicons were detected in 13 isolates (59%). Strains BPSAC26, BPSAC32, BPSAC33, BPSAC34, BPSAC35, BPSAC36, BPSAC37, BPSAC38, BPSAC39, and BPSAC42 showed positive amplification products with both the PKSI and NRPS primers (Table 2). Isolate no. BPSAC32 with limited antagonistic activity showed the presence of both PCR products. Two isolates (BPSAC24 and BPSAC30) showing a positive result against NRPS primers exhibited significant antimicrobial activities against all tested pathogens. These results indicate that some of the pathways encoding antimicrobial genes may not be functional under studied climatic or environmental conditions and can synthesize them if cultivated under the modified conditions. However, highest detections rates were found among the members of *Streptomyces* sp. where PKSI and NRPS amplification were observed in 53.3 and 66.6% of the strains, respectively. *Leifsonia xyli* (BPSAC24) and *Brevibacterium* sp. (BPSAC35), considered as rare genera among actinomycetes, also showed some biosynthetic potential. This knowledge may prove to be useful for isolation of natural products.

### Sequence alignment and phylogenetic analysis

**Figure 5:**
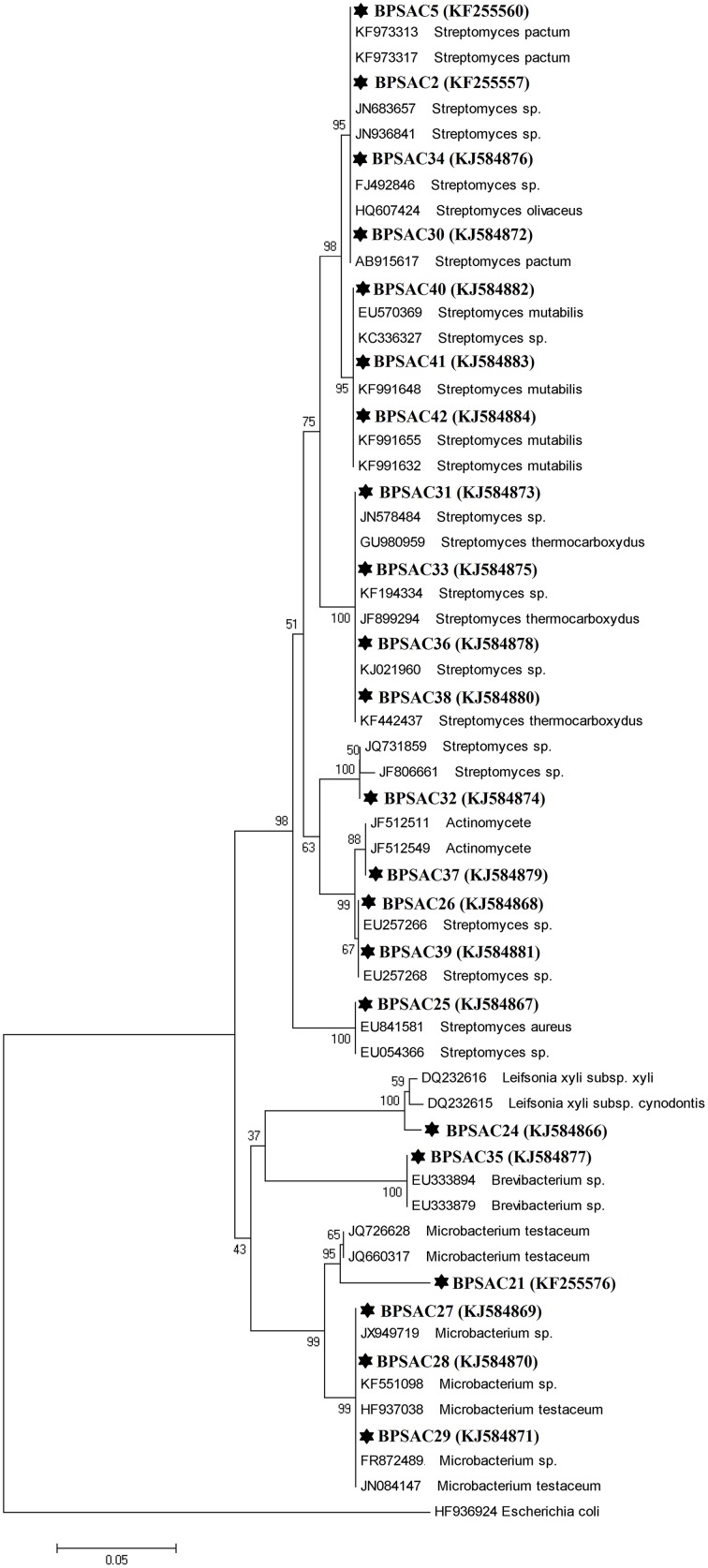
**Neighbor-joining phylogenetic tree based on 16S rRNA gene of endophytic actinomycetes**. Numbers at branches indicate bootstrap values of neighbor joining analysis (>50%) from 1000 replicates.

**Figure 6:**
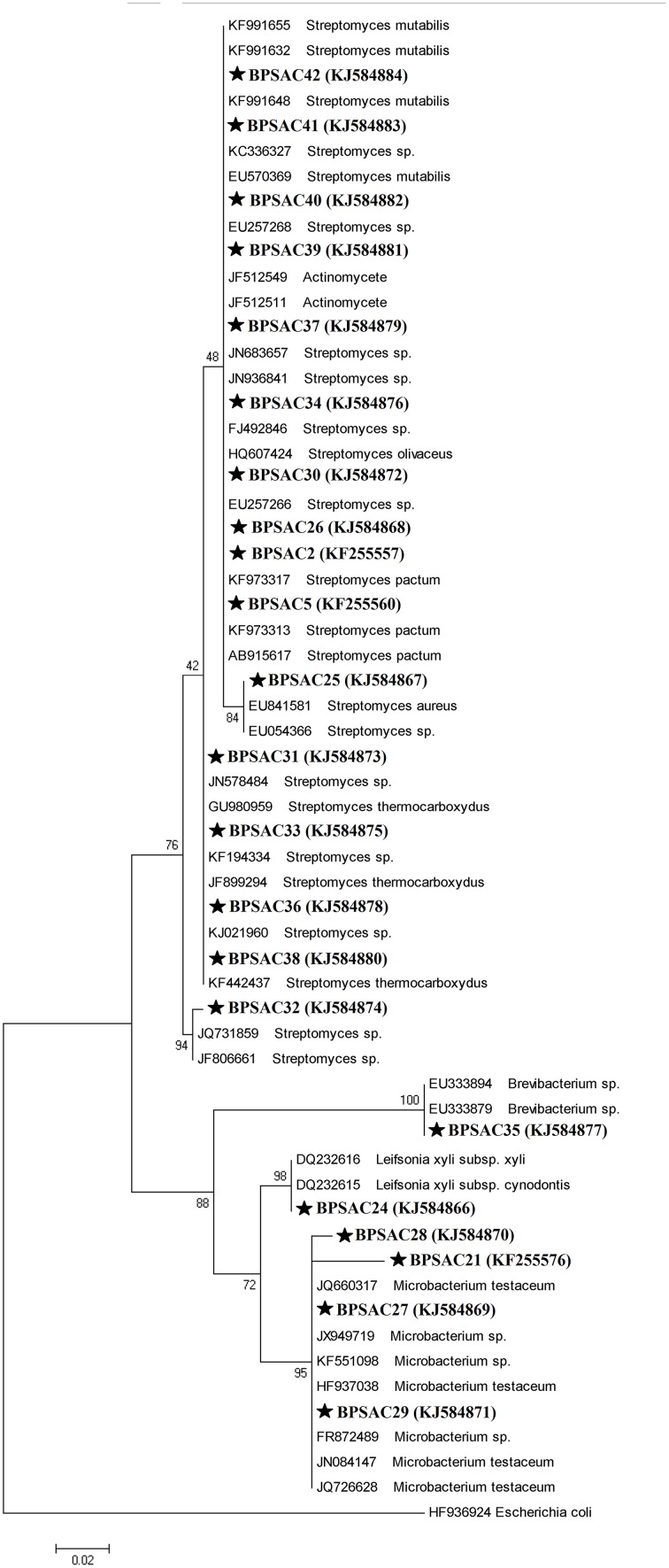
**Maximum likelihood phylogenetic tree based on 16S rRNA genes of endophytic actinomycetes from Mizoram**. Numbers at branches indicate Bootstrap values of neighbor-joining analysis (>50%) from 1000 replicates.

To investigate the relationships among the more promising endophytic actinomycetes isolate, 16S rRNA gene sequences were aligned along with the sequences of type strains retrieved from DDBJ/EMBL/NCBI GenBank databases. The results showed that the isolates were classified into four families and five genera. Most of the isolates grouped into *Streptomycetaceae* (68.18%), followed by *Microbacteriaceae* (22.7%), *Brevibacteriaceae*, and *Actinomycetaceae* (4.5% each). Analysis of the 16S rRNA gene sequence by BlastN with 99–100% similarity confirmed that 15 isolates could be members of genus *Streptomyces*. The sequences of the 4 isolates (BPSAC21, BPSAC27, BPSAC28, and BPSAC29) showed 96–100% identity to the sequences retrieved from genus *Microbacterium* and isolates BPSAC24, BPSAC35, and BPSAC37 showed high identity (99% each) to the genus *Leifsonia*, *Brevibacterium*, and *Actinomycete*, respectively (Table 3). Maximum-likelihood and neighbor-joining methods were used for the construction of phylogenetic tree. The topology of the phylogenetic tree generated by both methods showed that all *Streptomyces* forms a major clade I, along with the type strains retrieved from databases with the exception to *Actinomycete*, which also falls in the same clade under a bootstrap support value of 76 and 98%. Most of the putative species in the genera *Brevibacterium*, *Leifsonia*, and *Microbacterium*, clustering to form another clade II. The neighbor-joining analysis did not cluster *Leifsonia* and *Microbacterium* together, though they belong to same family, but maximum-likelihood clearly clusters both genera together (Figures 5, 6).

**Table 3:** **Identification of antagonistic potential endophytic actinomycetes based on 16S rRNA gene sequences**.

**Isolate No**	**NCBI-GenBank accession number**	**Closest species with accession number**	**Similarity**	**Identification**
BPSAC2	KF255557	*Streptomyces pactum*(KF973317)	99%	*Streptomyces* sp.
		*Streptomyces pactum*(KF973313)		
BPSAC5	KF255560	*Streptomyces pactum*(KF973313)	100%	*Streptomyces* sp.
		*Streptomyces pactum*(AB915617)		
BPSAC21	KF255576	*Microbacterium* *testaceum*(JQ726628) *Microbacterium* *testaceum*(JQ660317)	96%	*Microbacterium* sp.
BPSAC24	KJ584866	*Leifsonia xyli*(DQ232616)	99%	*Leifsonia xyli*
		*Leifsonia xyli*(AE016822)		
BPSAC25	KJ584867	*Streptomyces aureus*(EU841581)	99%	*Streptomyces* sp.
		*Streptomyces* sp.(EU054366)		
BPSAC26	KJ584868	*Streptomyces* sp.(EU257268)	99%	*Streptomyces* sp.
		*Streptomyces* sp.(EU257266)		
BPSAC27	KJ584869	*Microbacterium* sp.(JX949719)	100%	*Microbacterium* sp.
		*Microbacterium testaceum*(JN084147)		
BPSAC28	KJ584870	*Microbacterium* sp.(KF551098)	99%	*Microbacterium* sp.
		*Microbacterium testaceum*(HF937038)		
BPSAC29	KJ584871	*Microbacterium* sp.(FR872489)	99%	*Microbacterium* sp.
		*Microbacterium testaceum*(JN084147)		
BPSAC30	KJ584872	*Streptomyces olivaceus*(HQ607424)	98%	*Streptomyces olivaceus*
		*Streptomyces* sp.(FJ492846)		
BPSAC31	KJ584873	*Streptomyces* sp.(JN578484)	99%	*Streptomyces thermocarboxydus*
		*Streptomyces thermocarboxydus*(GU980959)		
BPSAC32	KJ584874	*Streptomyces* sp.(JQ731859)	99%	*Streptomyces* sp.
		*Streptomyces* sp.(JF806661)		
BPSAC33	KJ584875	*Streptomyces* sp.(KF194334)	99%	*Streptomyces* sp.
		*Streptomyces thermocarboxydus* (JF899294)		
BPSAC34	KJ584876	*Streptomyces* sp.(JN936841)	99%	*Streptomyces* sp.
		*Streptomyces* sp.(JN683657)		
BPSAC35	KJ584877	*Brevibacterium* sp.(EU333894)	99%	*Brevibacterium* sp.
		*Brevibacterium* sp.(EU333879)		
BPSAC36	KJ584878	*Streptomyces* sp.(KJ021960)	99%	*Streptomyces thermocarboxydus*
		*Streptomyces thermocarboxydus*(KF442437)		
BPSAC37	KJ584879	*Actinomycete*(JF512511)	99%	*Actinomycete*
		*Actinomycete*(JF512549)		
BPSAC38	KJ584880	*Streptomyces* sp.(KJ021960)	100%	*Streptomyces thermocarboxydus*
		*Streptomyces thermocarboxydus*(KF442437)		
BPSAC39	KJ584881	*Streptomyces* sp.(EU257268)	99%	*Streptomyces* sp.
		*Streptomyces* sp.(EU257266)		
BPSAC40	KJ584882	*Streptomyces mutabilis* (EU570369)	99%	*Streptomyces mutabilis*
		*Streptomyces* sp.(KC336327)		
BPSAC41	KJ584883	*Streptomyces mutabilis* (KF991655)	100%	*Streptomyces* sp.
		*Streptomyces mutabilis* (KF991648)		
BPSAC42	KJ584884	*Streptomyces mutabilis* (KF991655)	99%	*Streptomyces mutabilis*
		*Streptomyces mutabilis* (KF991632)		

## Discussion

Endophytic microorganisms acquire specific traits that allow them to thrive within the living tissues of a plant without any detectable infectious symptoms to the host. However, they are of immense importance due to their capability to produce wide array of natural bioactive compounds (Faeth and Hammon, 1997; Strobel and Long, 1998). Endophytic actinomycetes from different medicinal plants are reported as major source of natural products with potential antimicrobial activity (Cao et al., 2004; Castillo et al., 2007). These findings encouraged us to explore traditional medicinal plants of Mizoram for understanding the endophytic actinomycetes community and their biosynthetic potential as antimicrobial agents.

### Isolation, distribution and relative abundance of endophytic actinomycetes

Among 42 endophytic actinomycetes obtained from seven medicinal plants, *Streptomyces* was the dominant genus (*n* = 28, 66.6% of all isolates), a finding consistent with other reports from different hosts showing the dominance of species within the *Streptomyces* genus (Coombs and Franco, 2003; Cao et al., 2004). Besides *Streptomyces* sp., other genera were also reported occasionally from medicinal plants like *Microbacterium* from *Maytenus austroyunnanesis* (Qin et al., 2012), *Leifsonia* from Ginseng roots (Qiu et al., 2007) and *Brevibacterium* was isolated from *Centella asiatica* and *Conyza canadensis* (Kim et al., 2012; Rakotoniriana et al., 2013) Therefore, it looks like that these reported rare genera are also able to associate endophytically with various hosts. The *Streptomyces* genus has an excellent track record for the discovery of secondary metabolites. Strobel and Daisy (2003) described plant selection as tactical, plants with an unusual location and biology with traditional ethnobotanical history should be chosen for isolating endophytes producing novel bioactive products. Endophytic actinomycetes were isolated from every tissue; however, isolate to tissue ratios was highest in root tissues (*n* = 22, 52.3%) followed by stem (*n* = 9, 21.4%), leaf (*n* = 6, 14.2%), flower (*n* = 3, 7.1%), and petiole (*n* = 2, 4.7%), which indicates that endophytic actinomycetes are most dominant in the root tissues. Our results are consistent with the findings of Taechowian et al. (Taechowisan et al., 2003), who stated that roots represent a good territory for endophytic actinomycetes by examining 5400 different tissues from 36 species of plants in Thailand and recovering 212 (64%) isolates from roots followed by leaves (*n* = 97, 29%) and stem (*n* = 21, 6%). Furthermore, more than double the number of endophytic actinomycetes was recovered from roots (55%) than from stem (24%) and leaves (22%) from 20 different *Azadirachta indica* trees growing in northern India by Verma et al. (2009). Many researchers isolated most endophytic actinomycetes from roots (Taechowisan and Lumyong, 2003; Cao et al., 2005). This may be due to the fact that actinomycetes present in rhizosphere can be transferred very easily to plant roots since roots are the site of water and nutrient uptake. Also, actinomycetes get access to the plant when the epidermal layer gets damaged due to side roots growing out from the existing roots (Sardi et al., 1992).

Out of seven plants selected for this study, to our best understanding isolation of endophytic actinomycetes was attempted first time from five plants viz. *Mirabilis jalapa*, *Clerodendrum colebrookianum*, *Eupatorium odoratum*, *Alstonia scholaris*, and *Musa superba.* Among them the maximum endophytic isolates (*n* = 12) were recovered from *Mirabilis jalapa.*

### Evaluation of antimicrobial activity

We detected significant antimicrobial activity of endophytic actinomycetes isolated in this study against both Gram positive and Gram negative bacteria. Similarly, earlier studies also reported on potential antimicrobial activity from extracts isolated from endophytic actinomycetes (Taechowisan et al., 2003; Verma et al., 2009). It has also been reported that endophytic actinomycetes obtained from non-medicinal plants possess a small percentage with antimicrobial potential (Taechowisan et al., 2003). In our finding more than half, altogether 22 isolates showed antimicrobial activities against at least two tested pathogens, which clearly shows that endophytic actinomycetes associated with medicinal plants can have a greater degree of antagonistic activities. Our results support the hypothesis that the medicinal properties of the plants could be partially due to the existence of endophytes in the host (Strobel et al., 1999). The presence of antagonistic activity against tested bacterial and yeast pathogens guaranteed further study to find potential antimicrobial compounds. For example isolate BPSAC38 identified as *Streptomyces thermocarboxydus* showed significant antimicrobial activity against *S. aureus* (14.8 mm). Similarly, isolates BPSAC28, 35, and 37 isolated from *M. jalapa* and *C. colebrookianum*, respectively showed significant antimicrobial activity against *E. coli*, *P. aeruginosa*, and *C. albicans*. Furthermore, antioxidant, antimicrobial, and hypertension activity has been reported for these isolates (Nath and Bordoloi, 1991; Verma et al., 2009; Akintobi et al., 2011). The findings from our study are in agreement with previously reports of Li et al. (2008), who stated that endophytic *Streptomyces* species isolated from pharmaceutical plants have antimicrobial and antitumor properties. Several other researchers also support these findings that endophytic actinomycetes recovered from medicinal plants having ethnobotanical history are potential candidates for the recovery of potential antimicrobial natural products (Sharma et al., 2001).

### Antibiotic sensitivity assay

Here we detected significant antibiotic resistance activity of endophytic actinomycetes. Among 22 isolates four of them (BPSAC 26, 35, 38, and 42) were resistant against penicillin G, vancomycin, nalidixic acid, ampicillin, and streptomycin and nine isolates showed resistance against four of the tested antibiotics. Interestingly, the isolates that showed the most resistance against antibiotics also showed antagonistic activity. Isolates BPSAC26, 35, and 38 which were the most potential antimicrobial isolates were resistant against five major antibiotics. The characteristic of antibiotic resistance in bacterial endophytes from *Andrographis paniculata* leaves was reported by Pal et al. (2012), which also includes *Micrococcus*, an actinobacteria. Gousterova et al. (2014) tested the biosynthetic abilities of 26 thermophilic actinobacteria and screened for their sensitivity against 12 antibiotics. Few reports are available for the antibiotic sensitivity profiling among the endophytic actinomycetes associated with medicinal plants. Antibiotic-resistant strains are common in the environment irrespective of the human use of antibiotics. With the presence of plasmids, antibiotic resistance can transfer horizontally from one bacterium to another and also between phylogenetically distant bacteria, which contributes to the well-known problem of antibiotic resistance (Khachatourians, 1998; Davison, 1999).

### Detection of PKS and NRPS genes in selected strains

To understand the biosynthetic potential of the isolates, detection of genes encoding polyketide synthases and nonribosomal peptide synthetases, responsible for the synthesis of most biologically active polyketide and peptide compounds have been broadly used for assessing biosynthetic potential of culturable and non-culturable microorganisms (Minowa et al., 2007). However, the antimicrobial potential of the culturable actinomycetes may only be assessed by screening of antimicrobial activity against desired pathogens. The number of isolates having antimicrobial property does not correlate with the percentage of isolates showing the presence of PKSI and NRPS genes and vice versa (Li et al., 2008; Qin et al., 2009). In this study most of the isolates showed the presence of genes that could encode PKSI and NRPS enzymes, which also showed antimicrobial activity against most of the tested pathogens. However, five isolates (BPSAC25, 27, 29, 31, and 41) that were negative for PKSI and NRPS genes, also demonstrated non-significant antimicrobial potential. Similarly, only three isolates (BPSAC2, 32, and 42) that had PKSI and NRPS genes based on PCR amplification showed very limited antimicrobial activity. These results indicate that either the isolates possess moderate antimicrobial activity that is ineffective against the pathogens tested, or the quantity of antimicrobials produced was low which fails to elicit antimicrobial action. Lack of amplification of PKSI and NRPS in some of the isolates may be due to absence of these genes (Hornung et al., 2007; Qin et al., 2009). Furthermore, not all NRPS genes are involved in the production of secondary metabolites, rather they may play role in iron metabolism or quorum sensing (Finking and Marahiel, 2004). Interestingly, most of the isolates identified as *Streptomyces* sp. demonstrated the presence of antimicrobial genes were positive for most of the tested pathogens. Similar findings were reported by a subset of the researchers (Baltz, 2006; Qin et al., 2009; Zhao et al., 2011).

### Sequence alignment and phylogenetic analysis

All antagonistic isolates were characterized by PCR amplification of the 16S rRNA gene. Sequences were analyzed to predict the diversity of the isolates and assign them molecular taxonomic unit. DNA sequences of most isolates showed 97–100% identity with a reference sequence in GenBank. All the isolated endophytic actinomycetes were classified into four families and five possible genera, indicates a good association of endophytic actinomycetes associated with medicinal plants. The composition of endophytic actinomycetes as revealed by phylogenetic trees was more diverse as compared to endophytic actinomycetes isolated from *Azadirachta indica* (Verma et al., 2009), medicinal plants and rainforests of China (Li et al., 2008; Zhao et al., 2011). All the *Streptomyces* isolates fall under one major clade with an exception of *Actinomycete* sp. morphologically similar with *Streptomyces* sp. and hence clustered together and rarer isolates *Leifsonia xyli*, *Brevibacterium* sp., and *Microbacterium* were clustered together formed another major cluster, consistent with the findings of previous studies (Zhao et al., 2011). Rare endophytic actinomycetes of the genera *Leifsonia* and *Brevibacterium*, were first time found to be endophytic by Qiu et al. (2007) and Qin et al. (2012) both studying Chinese medicinal plants for the isolation of endophytes. Since, similar media and methods for the recovery of isolation were used in other studies, the findings of Qin et al. (2012), and our results indicates that in general medicinal plants harbors a good population of endophytic actinomycetes including some rare genera. This is the first report of *L. xyli* (BPSAC24) [KJ584866] isolated as an endophyte from the medicinal plant *C. colebrookianum*, though, Qiu et al. (2007) isolated *Leifsonia ginseng* from roots of medicinal plant *Panax ginseng*. It is also worth noting that *Brevibacterium* sp. isolate BPSAC35, recovered from stem of *M. jalapa* is the first from this genus to be reported as endophytic. The nucleotide sequences of 16S rRNA gene of all endophytic actinomycetes have been deposited in GenBank with accession number (KF255557, KF255560, KF255576, and KJ584866–KJ584884).

### Conflict of interest statement

The authors declare that the research was conducted in the absence of any commercial or financial relationships that could be construed as a potential conflict of interest.
